# TP53-centric ctDNA complements PET/CT for non-invasive assessment of pathological complete response and survival after neoadjuvant immunochemotherapy in esophageal squamous cell carcinoma: a prospective cohort study

**DOI:** 10.1097/JS9.0000000000002341

**Published:** 2025-03-28

**Authors:** Weixiong Yang, Si-Cong Ma, Zengli Fang, Yao Liu, Xin Zhang, Fang Wang, Chenxuan Wang, Yuze Wang, Xiaoyan Wang, Wenfang Chen, Hui Luo, Lingling Yang, Shuishen Zhang, Bo Zeng, Zhenguo Liu, Qiuxiang Ou, Junchao Cai, Sai-Ching Jim Yeung, Chao Cheng

**Affiliations:** a Department of Thoracic Surgery, The First Affiliated Hospital of Sun Yat-sen University, Guangzhou, Guangdong, China; b Institute of Precision Medicine, The First Affiliated Hospital of Sun Yat-sen University, Guangzhou, Guangdong, China; c Nanjing Geneseeq Technology Inc, Nanjing, Jiangsu, China; d Department of Nuclear Medicine, The First Affiliated Hospital of Sun Yat-sen University, Guangzhou, Guangdong, China; e Department of Pathology, The First Affiliated Hospital of Sun Yat-sen University, Guangzhou, Guangdong, China; f Zhongshan School of Medicine, Sun Yat-Sen University, Guangzhou, Guangdong, China; g Department of Emergency Medicine, The University of Texas MD Anderson Cancer Center, Houston, Texas

**Keywords:** ^18^F-FDG PET/CT, circulating tumor DNA (ctDNA), esophageal squamous cell carcinoma (ESCC), neoadjuvant immunochemotherapy (NICT), pathologic complete response (pCR), TP53

## Abstract

**Background:**

Accurate assessment of pathologic complete response (pCR) after neoadjuvant immunochemotherapy (NICT) is crucial to implement active surveillance or tailor therapeutic strategies for esophageal squamous cell carcinoma (ESCC), while reliable non-invasive methods for pCR prediction are lacking. We aimed to evaluate the potential of integrating circulating tumor DNA (ctDNA) and PET/CT for predicting pCR to NICT for ESCC.

**Methods:**

A total of 123 eligible patients were enrolled, including 68 patients from our prospective clinical trial (ChiCTR2000028900) and a real-world study (NCT04822103) that formed the discovery cohort, as well as 55 patients from another clinical trial (ChiCTR2100051763) comprising the validation cohort. Blood samples for ctDNA sequencing and PET/CT metrics were collected before and after NICT.

**Results:**

The ctDNA status and PET/CT parameters at the post-NICT stage rather than the pre-NICT stage significantly differentiated pCR from non-pCR patients. ctDNA and PET/CT synergistically enhanced the prediction of pCR from perspectives of sensitivity and specificity, respectively. The model integrating ctDNA concentration and mean standardized uptake value (SUVmean) demonstrated area under curves (AUCs) of 0.860 in the discovery cohort and 0.798 in the validation cohort for pCR prediction and stratified patients into high- and low-risk groups with differential survival prospects. The key gene modules converged on *TP53* as the core mutation for pCR prediction, among which those located in the exon regions contributed the most to its predictive capacity. The model constructed based on *TP53* mutation and SUVmean differentiated pCR from non-pCR with comparable performance to the model based on PET/CT and the overall ctDNA concentration.

**Conclusion:**

The combination of post-treatment *TP53*-centric ctDNA and PET/CT synergistically enhances the prediction of pCR following NICT in ESCC patients, indicating the potential to inform clinical decision-making for these patients.

## Introduction

Building on the success of immunochemotherapy as a primary treatment for advanced esophageal squamous cell carcinoma (ESCC)^[^1^]^, recent studies have demonstrated that neoadjuvant immunochemotherapy (NICT) exhibits significant antitumor efficacy, achieving a high pathological complete response (pCR) rate of 25–40%^[^2,3^]^. A randomized phase 3 clinical trial (ESCORT-NEO/NCCES01) demonstrated that NICT significantly increased the pCR rate compared to chemotherapy alone (28% vs 4.7%) for locally advanced ESCC, with a tolerable safety profile^[^4^]^. The high pCR rate is associated with longer disease-free survival (DFS) and overall survival (OS) durations^[^5^]^. Accurate assessment of pCR may facilitate organ preservation, thereby potentially avoiding unnecessary surgery and its associated morbidity^[^1,6^]^. Accurately identifying poor responders is also beneficial for timely adjustments to therapeutic strategies^[^7^]^. However, restaging after neoadjuvant therapy is considered a major challenge due to the difficulty in interpreting the radiological appearance of tumors and treated positive lymph nodes, which are affected by induced fibrosis and ulceration^[^8,9^]^. Therefore, selecting patients who achieve pCR after NICT is an unmet need in clinical practice.

Endoscopic biopsy and endoscopic ultrasound with fine-needle aspiration (EUS-FNA) enable tissue sampling, but they are invasive and have a substantial false-negative rate^[^10^]^. Considering the risk of complications and limited universal applicability due to high technical demands, biopsy is optional if surgery is planned after neoadjuvant therapy^[^1^]^, underscoring the importance of reliable non-invasive alternatives. Currently, fluorine 18 (^18^F)-fluorodeoxyglucose (FDG) positron emission tomography/computed tomography (PET/CT) is widely recommended as a non-invasive method to assess changes in tumor metabolic activity and tumor viability by measuring the intensity of FDG uptake, independent of underlying structural changes^[^11,12^]^; however, its predictive efficacy for lymph node metastasis is limited by a high false-positive rate of 66.6%^[^13^]^. These findings highlight a critical need for a more effective and non-invasive method to predict pCR for NICT in patients. In recent years, circulating tumor DNA (ctDNA) sequencing has emerged as a non-invasive method for early diagnosis, prognostic stratification, disease surveillance, and treatment response evaluation across different cancer types^[^14^]^. Given its convenient, real-time, and non-invasive features, ctDNA can monitor cancer evolution and therapeutic effects in real time, thus effectively guiding personalized treatment and evaluating treatment response^[^15,16^]^. Despite these advantages, evidence is lacking to support the effectiveness of ctDNA for evaluating the pathological response to the NICT in ESCC patients.

The aim of this study was to explore the combination of non-invasive ctDNA and PET/CT assessments in predicting the pathological response and survival of NICT in patients with locally advanced ESCC. We hypothesized that this combined approach would improve the accuracy of pCR and survival prediction in these patients. This exploratory study included cohorts from our previous prospective clinical trial (ChiCTR2000028900) and real-world study (NCT04822103), both of which focused on neoadjuvant PD-1 blockade in combination with chemotherapy for resectable ESCC. Additionally, we included a cohort from another prospective clinical trial (ChiCTR2100051763) for independent validation analysis.

## Methods

### Patients and study design

The discovery cohort consisted of 68 patients from our prospective clinical trial (ChiCTR2000028900, registered at Chinese Clinical Trial Registry, http://www.chictr.org.cn/, *n* = 20) and a real-world study (NCT04822103, registered at ClinicalTrials.gov, https://clinicaltrials.gov, *n* = 48). Eligible patients with potentially resectable ESCC at clinical TNM stages II–III were recruited from the First Affiliated Hospital of Sun Yat-Sen University. The enrolled patients underwent an NICT regimen, comprising two cycles of the anti-PD-1 antibody (camrelizumab) in combination with nab-paclitaxel and carboplatin. The validation cohort consisted of 55 patients from another phase II clinical trial (ChiCTR2100051763, registered at Chinese Clinical Trial Registry, http://www.chictr.org.cn/). Patients with untreated, resectable (stage II or III) ESCC were enrolled, and each patient also received an NICT regimen. All enrolled patients were planned for surgery, so endoscopic biopsy before surgery was optional^[^1^]^. After the surgery, patients were followed up every 3 months for the first 2 years and then every 6 months for up to 5 years. OS was defined as the time duration from the date of diagnosis of ESCC to the date of death from any cause. DFS was defined as the time duration from the date of surgery to the date of recurrence. This study was approved by the Institutional Ethics Committee for Clinical Research and Animal Trials of the First Affiliated Hospital of Sun Yat-Sen University (approval ID: [2022]188). All patients provided written informed consent. The work was reported in line with the strengthening the reporting of cohort, cross-sectional, and case–control studies in surgery (STROCSS) criteria^[^17^]^.

**HIGHLIGHTS**

- ctDNA complements PET/CT for non-invasive assessment of pathologic complete response (pCR) for neoadjuvant immunochemotherapy in esophageal squamous cell carcinoma.
- The predictive model integrating ctDNA concentration and mean standardized uptake value (SUVmean) not only assesses the efficacy of the treatment but also prognosticates patient outcomes.
- *TP53* was identified as the core ctDNA mutation for pCR prediction.
- *TP53*-targeted ctDNA detection offers a more cost-effective option for patients.

Pathological response was assessed and confirmed by consensus of two blinded pathologists. According to previously reported methods^[^18^]^, immune-related pathologic response criteria (irPRC) were applied to assess pathological response using the percentage of immune-related residual viable tumor (%irRVT). Specifically, %irRVT = viable tumor area/total tumor bed area, whereby the total tumor bed = regression bed + residual viable tumor (RVT) + necrosis. Pathological complete response (pCR) was defined as the absence of viable tumor cells in the resected cancer specimen without accompanying lymph node metastasis; major pathological response (MPR) was defined as the presence of ≤10% viable tumor cells in the resected cancer specimen; pathological partial response (pPR) was defined as the presence of >10% but ≤50% viable tumor cells in the resected cancer specimen; pathological stable disease (pSD) was defined as the presence of >50% viable tumor cells in the resected cancer specimen. In this study, MPR, pPR, and pSD were defined as incomplete pathological responses.

### ^18^F-fluorodeoxyglucose (FDG) PET/CT imaging and analysis

Patients were instructed to perform PET/CT examinations at pre- and post-NICT (before surgery).^18^F-FDG PET/CT imaging was performed as described in our previous study^[^13^]^. After images were obtained, we used PET volume computer-assisted reading (PETVCAR) system to analyze the images. The metabolic parameters calculated by PETVCAR include maximum standardized uptake value (SUVmax), mean standardized uptake value (SUVmean), metabolic tumor volume (MTV), and total lesion glycolysis (TLG). Subsequently, the ratio of SUVmax of primary tumor to SUVmax of blood pool (SUVTBR) was calculated.

### Sequence data processing and identification of clinically actionable mutations

Blood samples were collected before the start of neoadjuvant treatment (mean time interval: 0.71 days, range 0–2 days) and before the surgery (mean time interval: 1.18 days, range 0–4 days) and then centrifuged within 24 hours of collection to separate the plasma and the sediment. Plasma was utilized to extract ctDNA for subsequent sequencing. Blood sediment was used to extract DNA from white blood cells (WBC), which was then sequenced to identify germline mutations. The DNA quality was assessed using Nanodrop2000 (Thermo Fisher Scientific), and the quantity was measured using a dsDNA HS Assay Kit (Life Technologies) on Qubit 2.0.

### Library preparation and sequencing

WBC DNA was fragmented into 300–350 bp using the Covaris M220 instrument (Covaris). Sequencing libraries were prepared with a KAPA Hyper Prep kit (KAPA Biosystems) with optimized protocols. In brief, plasma ctDNA and WBC DNA were processed through end-repairing, A-tailing, adapter ligation, and size selection using Agencourt AMPure XP beads (Beckman Coulter). Libraries were then subjected to PCR amplification and purification before targeted enrichment.

DNA libraries from different samples were marked with unique indices during library preparation, and up to 2 μg of different libraries were pooled together for targeted enrichment. A customized xGen lockdown probes panel (Integrated DNA Technologies) was used to target enrichment for 196 predefined genes. The list of 196 predefined genes included in ctDNA mutation profiling was provided in Supplementary Table S1 http://links.lww.com/JS9/E32. The hybridization reaction was performed using the NimbleGen SeqCap EZ Hybridization and Wash Kit (Roche). Dynabeads M-270 (Life Technologies) was used to capture probe-bind fragments, followed by library amplification with Illumina p5 in KAPA HiFi HotStart ReadyMix (KAPA Biosystems) and purification using Agencourt AMPure XP beads. The size distribution of libraries was measured by the Agilent Technologies 2100 Bioanalyzer (Agilent Technologies). The enriched libraries were sequenced on NovaSeq 6000 NGS platforms (Illumina). The ctDNA samples were sequenced at a depth of 30 000×, while the WBC samples were sequenced at a depth of 250×.

Trimmomatic was used for FASTQ file quality control. Leading or trailing low quality (quality reading below 15) or N bases were removed. Reads from each sample were mapped to the reference sequence hg19 (Human Genome Version 19) using Burrows-Wheeler Aligner (BWA-mem, v0.7.12) with parameters (-t 8 -M). Local realignment around insertions and deletions (indels) and base quality score recalibration were applied with the Genome Analysis Toolkit (GATK 3.4.0). GATK3.4.0 was applied to detect germline mutations in control blood samples. VarScan2 was employed for the detection of somatic mutations (somatic *P* value = 0.1, minimum quality score = 15, and otherwise default parameters). Somatic variants were analyzed using Automated Triple Groom Sequencing (ATG-Seq) technology^[^19^]^. Annotation was performed using ANNOVAR using the hg19 reference genome and 2014 versions of standard databases and functional prediction programs. Relative ctDNA abundance was calculated by multiplying the maximum value of variant allele frequency (VAF) by the cell-free DNA (cfDNA) concentration (ng/ml)^[^20^]^. ctDNA concentration (hGE/ml) was calculated by multiplying the mean value of VAF by the cfDNA concentration (pg/ml) and dividing by haploid genomic equivalent weighs 3.3 pg^[^21^]^. At each time point, gene mutations with VAF ≥ 0.25% were classified as positive.

### Model development and evaluation

Least absolute shrinkage and selection operator (LASSO) regression was used for preliminary feature selection using the R package “glmnet,” followed by stepwise regression in both directions to remove collinear variables using the R package “stats” with the “step” function. Logistic regression was used to integrate ctDNA and PET/CT metrics for the prediction of pCR status. Area under receiver operating characteristic (ROC) curves (AUCs) with 95% confidence intervals (CIs) were calculated to evaluate the predictive capacity for the pCR status of a specific variable or model score; leave-one-out cross-validation (LOOCV) was used in the discovery cohort, followed by external testing in the validation cohort. The DeLong test was used to compare the AUCs of paired ROCs using the R package “pROC.” The sensitivity and specificity were calculated for ctDNA and PET/CT metrics, with the optimal cutoff determined by Youden index. Given that pCR patients account for about one-third of the population, the upper one-third of the model score was used as the cut point to categorize patients into low- and high-risk groups. The detailed formulas and cutoff values of the prediction model integrating ctDNA concentration and SUVmean were as follows: Model score = −0.9858 × SUVmean − 0.0109 × ctDNA_concentration; Cutoff (training + validation) = −1.158552 − 1.5030 = −2.6616; Low-risk: model score ≤−2.6616; High-risk: model score >−2.6616. More details of the prediction models used in this article were provided in the Supplementary materials, Supplemental Digital Content, http://links.lww.com/JS9/E32.

### Generation of ctDNA modules

The 196 predefined genes included in ctDNA mutation profiling were clustered into modules from the perspectives of both protein–protein interaction (PPI-based ctDNA modules) and treatment response (treatment-based ctDNA modules), respectively. A total of six PPI-based ctDNA modules (P1–P6) were generated using Metascape (https://metascape.org/) for protein–protein interaction enrichment analysis with a minimum network size of 3 and a maximum network size of 500, while six treatment-based ctDNA models (T1–T6) were generated based on the established role of each gene mutation for different treatment paradigms.

### Statistical analyses

The Wilcoxon rank sum test was used to compare two groups on a continuous variable. The Fisher’s exact test was used to assess the significance of an association between two categorical variables. The survival curves were generated using the Kaplan–Meier method and compared using the log-rank test. A *P* value of less than 0.05 was considered statistical significance. Statistical analyses were conducted using R (version 4.1.1) and GraphPad Prism (version 8).

## Results

### Patient characteristics

**Figure 1.**
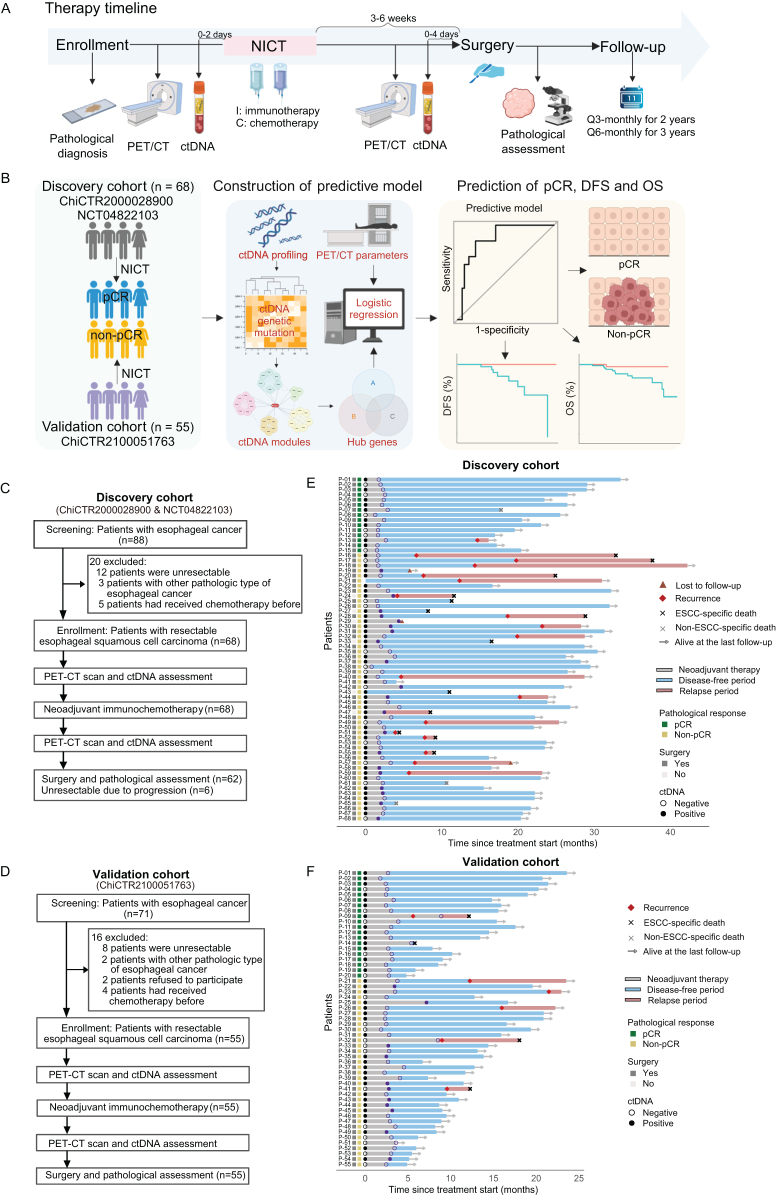
Study overview. (A) Therapy timeline, sample collection, and follow-up duration in patients with esophageal cancer. All eligible ESCC patients received NICT, followed by surgery. Peripheral blood samples were collected for ctDNA assessment before the start of neoadjuvant treatment (range 0–2 days) and before the surgery (range 0–4 days). After the surgery, patients were followed up every 3 months for 2 years and then every 6 months for up to 5 years. (B) Study design: 68 patients from our prospective clinical trial (ChiCTR2000028900) and a real-world study (NCT04822103) formed the discovery cohort and 55 patients from another clinical trial (ChiCTR2100051763) comprised the validation cohort. The predictive model was constructed based on ctDNA and PET/CT parameters to predict the pCR, DFS, and OS of patients. (C and D) Flow diagrams of the discovery and validation cohorts. (E and F) Swimmer plots depict the length of follow-up and events for each evaluable patient in the (E) discovery and (F) validation cohorts. DFS, disease-free survival; NICT, neoadjuvant immunochemotherapy; OS, overall survival; pCR, pathologic complete response; PET/CT, positron emission tomography/computed tomography.

All eligible ESCC patients received two or three 21-day cycles of NICT followed by surgery. Peripheral blood samples were collected before the start of neoadjuvant treatment (mean time interval: 0.71 days, range 0–2 days) and before the surgery (mean time interval: 1.18 days, range 0–4 days; Fig. 1A). The discovery cohort consisted of 68 patients from a clinical trial (ChiCTR2000028900) and a real-world study (NCT04822103), and the validation cohort included 55 patients from another clinical trial (ChiCTR2100051763; Fig. 1B). We constructed a predictive model based on ctDNA and PET/CT parameters collected after NICT to predict the pCR, disease-free survival (DFS) and overall survival (OS). Hub gene mutations predictive of pCR were identified through construction and analyses of ctDNA mutation modules.

From March 2020 to February 2022, 88 patients with ESCC were screened for eligibility (Fig. 1C). Among them, 20 patients were excluded because of the initial unresectable clinical stage, a non-ESCC pathological type, or prior chemotherapy. Consequently, 68 eligible patients were enrolled in the discovery cohort and received NICT. Sixty-two patients underwent surgery, but six patients were unresectable due to disease progression. Residual tumors were confirmed in all six patients through biopsy. In the validation cohort, 71 ESCC patients were screened, but 16 patients were excluded due to the initial unresectable tumor, non-ESCC pathological type, prior chemotherapy, or refusal to participate. As a result, 55 eligible patients were enrolled, and all of them received the NICT and surgery (Fig. 1D).

The baseline clinical characteristics are shown in Table 1. In the discovery cohort, 52.9% of patients were under 60 years old, and the majority were male (79.4%). Of the 68 patients, 29 patients (44.6%) were diagnosed with stage II, 32 patients (47.1%) with stage III, and 7 patients (10.3%) with stage IV. After NICT, surgery was performed in 62 patients (91.2%), and pCR was achieved in 15 patients (22.1%), according to the pathological assessment of the surgical specimens. No significant differences were observed in baseline clinical characteristics between the discovery and validation cohorts. Swimmer plots depicted the length of follow-up and events for each evaluable patient in the discovery and validation cohorts (Fig. 1E and F, respectively).

**Table 1:** Comparison of the clinicopathological characteristics between the discovery and validation cohorts

Characteristics	Discovery cohort	Validation cohort	*P* value
*n* (%)	(*n* = 68)	(*n* = 55)	
Age (years)			0.365
<60	36 (52.9)	24 (43.6)	
≥60	32 (47.1)	31 (56.4)	
Sex			1
Male	54 (79.4)	43 (78.2)	
Female	14 (20.6)	12 (21.8)	
Smoking history			0.147
Yes	34 (50)	35 (63.6)	
No	34 (50)	20 (36.4)	
Alcohol history			0.104
Yes	29 (42.6)	32 (58.2)	
No	39 (57.4)	23 (41.8)	
Tumor location in esophagus			0.586
Upper & Middle	34 (50)	24 (43.6)	
Distal	34 (50)	31 (56.4)	
Clinical T stage			0.786
T2	9 (13.2)	6 (10.9)	
T3-4	59 (86.8)	49 (89.1)	
Clinical N stage			0.355
N0	30 (44.1)	19 (34.5)	
N1–N3	38 (55.9)	36 (65.5)	
Clinical AJCC stage			0.855
II	29 (42.6)	22 (40)	
III and IV	39 (57.4)	33 (60)	
Pathological AJCC stage			0.244	
I	44 (64.7)	33 (60)		
II–IV	18 (35.3)	22 (40)		
Lymph node metastasis after NICT			0.534	
Yes	15 (22.1)	17 (30.9)		
No	47 (77.9)	38 (69.1)		
Numbers of dissected lymph node			0.362	
<22	15 (22.1)	9 (16.4)		
≥22	47 (77.9)	46 (83.6)		
Pathological response			0.304	
pCR	15 (22.1)	17 (30.9)		
MPR	19 (27.9)	15 (27.3)		
pPR	20 (29.4)	18 (32.7)		
pSD	14 (20.6)	5 (9.1)		

AJCC, the American Joint Committee on Cancer; NICT, neoadjuvant immunochemotherapy; pCR, pathological complete response; MPR, major pathological response; pPR, pathological partial response; pSD, pathological stable disease.

### Post-NICT ctDNA and PET/CT parameters instead of pre-NICT forecast pCR

**Figure 2.**
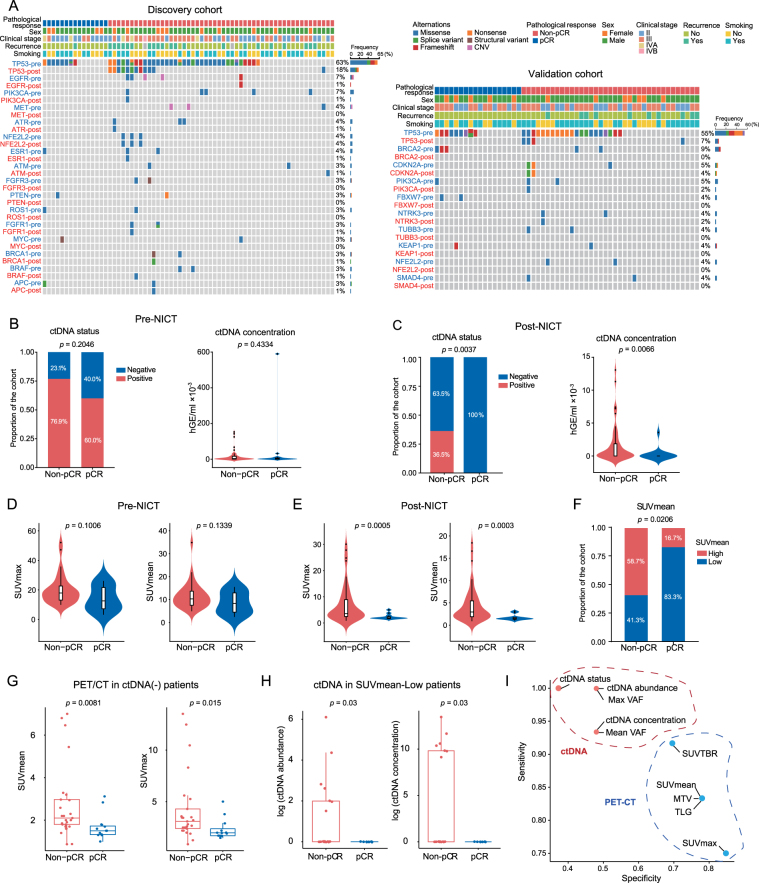
ctDNA and PET/CT synergistically predict pCR. (A) Distribution of genetic variation in pre- and post-NICT ctDNA genotyping analyses in the discovery and validation cohorts. (B and C) Comparison of pre-NICT (B) and post-NICT (C) ctDNA parameters between non-pCR and pCR patients in the discovery cohort. (D and E) Comparison of pre-NICT (D) and post-NICT (E) PET/CT parameters between non-pCR and pCR patients in the discovery cohort. (F) Comparison of post-NICT SUVmean status between non-pCR and pCR patients. (G) Comparison of post-NICT SUVmean and SUVmax values between non-pCR and pCR patients with negative ctDNA. (H) Comparison of post-NICT ctDNA abundance and concentration between non-pCR and pCR patients with SUVmean-Low patients. (I) The sensitivity and specificity of ctDNA and PET/CT parameters in predicting pCR. *P* values were determined by Wilcoxon rank sum test (for continuous variables) or Fisher’s exact test (for categorical variables). MTV, metabolic tumor volume; pCR, pathologic complete response; SUVmax, maximum standardized uptake value; SUVmean, mean standardized uptake value; SUVTBR, the ratio of SUVmax of primary tumor to SUVmax of blood pool; TLG, total lesion glycolysis; VAF, variant allele frequency.

In the discovery cohort, ctDNA was detected in 51 (75%) and 21 (30.9%) patients pre- or post-NICT, respectively. Before NICT, the most frequently altered genes were *TP53* (63%, *n* = 43), *EGFR* (7%, *n* = 5), *PIK3CA* (7%, *n* = 5), *MET* (4%, *n* = 3), *ATR* (4%, *n* = 3), *NFE2L2* (4%, *n* = 3), and *ESR1* (4%, *n* = 3) (Fig. 2A and Supplementary Fig. S1A, Supplemental Digital Content, http://links.lww.com/JS9/E32). After NICT, the most frequently altered genes were *TP53* (18%, *n* = 12) and *NFE2L2* (4%, *n* = 3). The mutation rate of *TP53* significantly decreased after NICT and cleared in all pCR patients (Fig. 2A and Supplementary Fig. S1B, Supplemental Digital Content, http://links.lww.com/JS9/E32). The ctDNA profile of the validation cohort was similar to that of the discovery cohort (Fig. 2A), with the most frequently altered genes of *TP53* (55%, *n* = 30), *BRCA2* (9%, *n* = 5), *CDKN2A* (5%, *n* = 3), *PIK3CA* (5%, *n* = 3), and *NFE2L2* (4%, *n* = 2). Most genomic changes in ctDNA were mutations (75%), followed by copy number variations (CNV) and structural variations (SV) with incidence rates of 8.82% and 5.88%, respectively (Supplementary Fig. S1C, Supplemental Digital Content, http://links.lww.com/JS9/E32). The most common co-mutated genes with *TP53* were *NFE2L2, EGFR*, and *PIK3CA* (Supplementary Fig. S1D, Supplemental Digital Content, http://links.lww.com/JS9/E32).

For predicting pCR, pre-NICT ctDNA (Fig. 2B) and PET/CT parameters (Fig. 2D and Supplementary Fig. S2A, Supplemental Digital Content, http://links.lww.com/JS9/E32) showed very limited predictive power. Remarkably, the post-NICT ctDNA status and concentration were associated with treatment efficacy (Fig. 2C), and all PET/CT metabolic parameters were significantly lower in pCR patients compared to those in the non-pCR counterparts (Fig. 2E and Supplementary Fig. S2B, Supplemental Digital Content, http://links.lww.com/JS9/E32). No significant difference was observed in pCR proportion between patients with cleared ctDNA and those with consistently negative ctDNA, indicating that the pCR predictive efficacy of post-NICT ctDNA is independent of pre-NICT ctDNA status (Supplementary Fig. S2C, Supplemental Digital Content, http://links.lww.com/JS9/E32). In the validation cohort, we also found that the post-NICT ctDNA concentration and PET/CT parameters were significantly lower in pCR patients compared to those with non-pCR, while pre-NICT parameters showed no difference between the groups (Supplementary Fig. S2D and E, Supplemental Digital Content, http://links.lww.com/JS9/E32). Additionally, the dynamic changes in ctDNA and PET/CT parameters before and after NICT showed no significant differences between the pCR and non-pCR groups both in the discovery cohort and the validation cohort (Supplementary Fig. S2F and G, Supplemental Digital Content, http://links.lww.com/JS9/E32).

### Post-NICT ctDNA and PET/CT synergistically contribute to pCR prediction on sensitivity and specificity

Analysis of the post-NICT ctDNA status and metabolic parameters showed that 63.5% of non-pCR patients were ctDNA-negative (Fig. 2C), and 41.3% of non-pCR patients had low SUVmean (Fig. 2F). These findings indicate that either ctDNA or PET/CT assessment alone cannot accurately evaluate whether patients have achieved complete remission after receiving NICT. Further analyses demonstrated that in the ctDNA-negative subgroup, non-pCR patients had higher SUVmean and SUVmax values compared to pCR patients (Fig. 2G). Conversely, in the SUVmean-Low subgroup, non-pCR patients had higher ctDNA abundance and concentration compared to pCR patients (Fig. 2H). In predicting treatment response, ctDNA parameters exhibited higher sensitivity, while PET/CT parameters showed higher specificity (Fig. 2I), suggesting that combining ctDNA with PET/CT may provide a more reliable and accurate predictive model.

### Predictive model for pCR based on non-invasive post-NICT ctDNA and PET/CT

**Figure 3.**
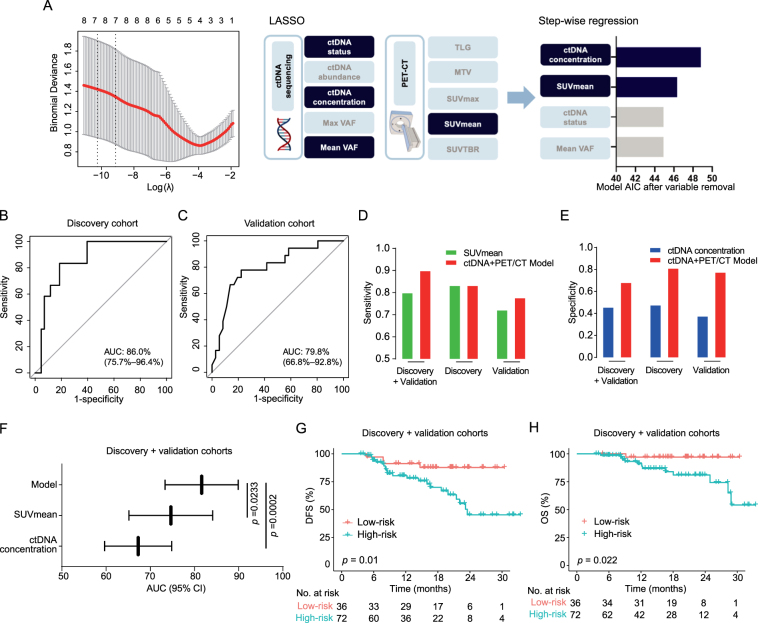
Construction of the pCR predictive model. (A) LASSO regression and stepwise regression were used to select variables for constructing pCR prediction model. (B and C) ROC reflecting the pCR predictive performance of the prediction model in the (B) discovery and (C) validation cohorts. (D) Comparison of the sensitivity between SUVmean and the ctDNA + PET/CT model for pCR prediction. (E) Comparison of the specificity between ctDNA concentration and the ctDNA + PET/CT model for pCR prediction. (F) Comparison of pCR predictive efficiency for the constructed model and SUVmean or ctDNA concentration alone. (G) Kaplan–Meier curves demonstrating the DFS outcomes of low-risk (red) and high-risk (green) patients in the combined cohort. (H) Kaplan–Meier curves demonstrating the OS outcomes of low-risk (red) and high-risk (green) patients in the combined cohort. *P* values in panel (F) were determined by DeLong test; *P* values in panel (G and H) were determined by Log-rank test. AIC, Akaike information criterion; AUC, area under curve; DFS, disease-free survival; LASSO, least absolute shrinkage and selection operator; MTV, metabolic tumor volume; OS, overall survival; SUVmax, maximum standardized uptake value; SUVmean, mean standardized uptake value; SUVTBR, the ratio of SUVmax of primary tumor to SUVmax of blood pool; TLG, total lesion glycolysis; VAF, variant allele frequency.

Given the complementary predictive capacity of ctDNA and PET/CT metrics for pCR, we attempted to explore the optimal combination of these two dimensions. Based on LASSO regression for feature selection and stepwise regression to avoid collinearity, ctDNA concentration and SUVmean emerged as the optimal combination parameters for pCR prediction (Fig. 3A), outperforming other pairwise combinations (Supplementary Fig. S3A, Supplemental Digital Content, http://links.lww.com/JS9/E32). The logistic regression model integrating ctDNA concentration and SUVmean showed satisfactory predictive performance (AUC = 0.860; 95% CI: 0.757–0.964) for the pCR status in ESCC patients using receiver operator characteristic (ROC) analysis (Fig. 3B). The effectiveness of the model was validated in the independent validation cohort (AUC = 0.798; 95% CI: 0.668–0.928; Fig. 3C). Moreover, the combination model had higher sensitivity than the SUVmean-only model and higher specificity than the ctDNA concentration-only model (Fig. 3D and E). Importantly, the constructed model had a significantly higher AUC than models using SUVmean (*P* = 0.0233) or ctDNA concentration (*P* = 0.0002) alone (Fig. 3F).

As for the long-term survival outcomes, ESCC patients who achieved pCR after receiving NICT had longer DFS (*P* = 0.00072, Supplementary Fig. S3B, Supplemental Digital Content, http://links.lww.com/JS9/E32) and OS (*P* = 0.016, Supplementary Fig. S3C, Supplemental Digital Content, http://links.lww.com/JS9/E32). Therefore, we investigated the prognostic value of the pCR prediction model. We calculated the risk scores of patients according to the model and divided them into high- and low-risk groups. Given that approximately one-third of the patients achieved pCR in both cohorts, we defined the low-risk group as the lowest tertile and the high-risk group as the upper and middle tertiles. Survival analysis showed that low-risk patients had longer DFS (Fig. 3G and Supplementary Fig. S3D and E, Supplemental Digital Content, http://links.lww.com/JS9/E32) and OS (Fig. 3H and Supplementary Fig. S3F and G, Supplemental Digital Content, http://links.lww.com/JS9/E32) compared to high-risk patients.

### Post-NICT exon mutations of TP53 contribute the most to pCR predictive efficiency

**Figure 4.**
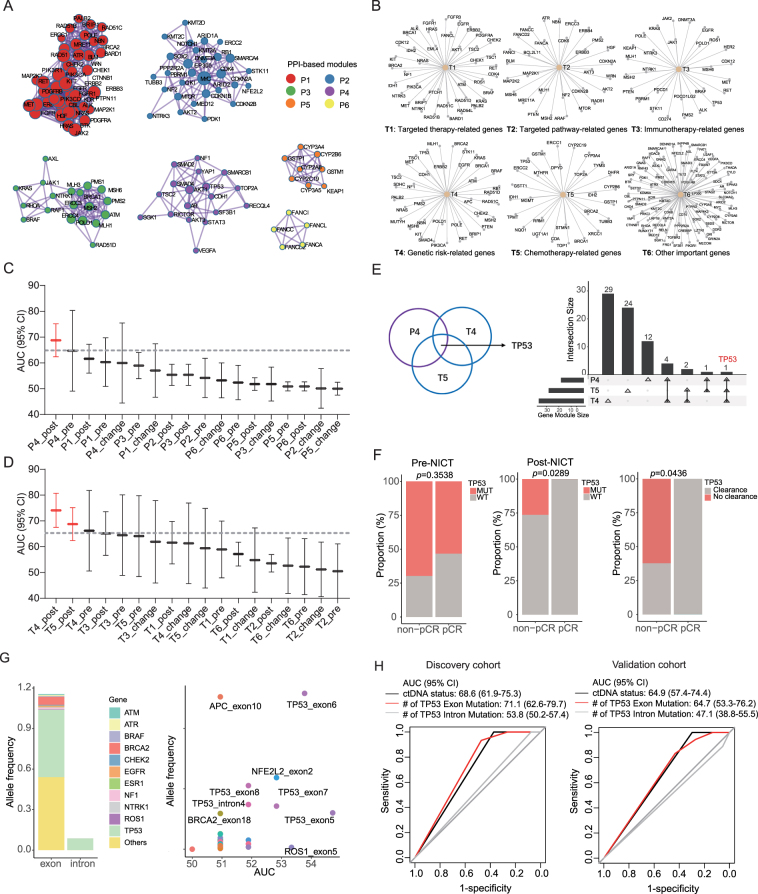
The exon mutations of *TP53* contribute the most to the pCR predictive efficiency. (A and B) Gene modules classified according to (A) protein–protein interaction network and (B) reported role for therapy response in different treatment paradigms. (C and D) The AUCs of different gene modules in predicting pCR. (E) Hub gene-*TP53* overlapped in P4, T4, and T5 modules. (F) Comparison of pre- and post-NICT *TP53* mutation status and TP53 clearance between non-pCR and pCR patients. (G) Summary of the frequency of gene exon and intron mutations in ctDNA and their AUCs for pCR prediction. (H) ROC reflecting the pCR predictive performance of *TP53* exon and intron mutations in the discovery and validation cohorts. *P* values in panel (F) were determined by Fisher’s exact test. #, number; AF, allele frequency; AUC, area under curve; PPI, protein–protein interaction.

To reverse engineer the model and interpret the capability of ctDNA in pCR prediction, we generated mutation modules from the ctDNA mutation profile. The ctDNA mutations were classified into gene modules according to the protein–protein interaction network (Fig. 4A) and their reported role for therapy response in different treatment paradigms, including immunotherapy, chemotherapy, and target therapy (Fig. 4B). As shown in Fig. 4C and D, post modules of P4, T4, and T5 had the highest AUC (>0.65) to discriminate pCR from patients with non-pCR. Genes overlapping in P4, T4, and T5 modules were defined as hub genes, which included *TP53* only (Fig. 4E). The post-NICT *TP53* mutation status and clearance were significantly associated with treatment efficacy (Fig. 4F). Subsequently, we verified that the post-NICT *TP53* mutation status had the optimal predictive performance compared to the pre-NICT *TP53*, the dynamic changes of *TP53*, and the mutation status of other genes (Supplementary Fig. S4A, Supplemental Digital Content, http://links.lww.com/JS9/E32). Notably, we found no differences between *TP53* allele frequency and *TP53* mutation status in predicting pCR (Supplementary Fig. S4B, Supplemental Digital Content, http://links.lww.com/JS9/E32), suggesting that it is the function of *TP53* that matters in pCR inference, instead of the types of mutations or their exact concentration. There was no significant difference in pCR proportion between the two groups of patients with mutation clearance or consistently negative *TP53* mutation status, indicating that the pCR predictive efficacy of post-NICT *TP53* mutation status is independent of the pre-NICT *TP53* mutation status (Supplementary Fig. S4C, Supplemental Digital Content, http://links.lww.com/JS9/E32). For external validation, we tested the predictive value of the overall ctDNA status and the ctDNA-based *TP53* mutation status in a recently published study on ESCC patients receiving definitive chemoradiotherapy combined with immunotherapy^[^22^]^ and found that the AUCs for ctDNA and *TP53* statuses were 0.707 and 0.612, respectively, which are close to the predictive values in our study (Supplementary Fig. S4D, Supplemental Digital Content, http://links.lww.com/JS9/E32).

We further explored the mutation regions in *TP53* with the greatest contribution. We found that the frequency of *TP53* exon mutations was significantly higher than that of introns (Fig. 4G and Supplementary Fig. S4E, Supplemental Digital Content, http://links.lww.com/JS9/E32). *TP53* exon 6 and *APC* exon 10 were the most frequent exons, but only *TP53* exon 6 has the highest AUC for pCR prediction (Fig. 4G). The frequency of *TP53* exon mutations in non-pCR patients is significantly higher than that in pCR patients (*P* = 0.0044 in the discovery cohort; *P* = 0.039 in the validation cohort; Supplementary Fig. S4E, Supplemental Digital Content, http://links.lww.com/JS9/E32). However, there is no significant difference in the frequency of *TP53* intron mutations between non-pCR and pCR patients (Supplementary Fig. S4F, Supplemental Digital Content, http://links.lww.com/JS9/E32). Further analysis indicated that mutations in exons 5, 6, and 7 of *TP53* had the optimal pCR predictive efficacy (Supplementary Fig. S4G, Supplemental Digital Content, http://links.lww.com/JS9/E32) and exerted synergistic effect as their combination increased the predictive capacity (Supplementary Fig. S4H and I, Supplemental Digital Content, http://links.lww.com/JS9/E32). The AUC of *TP53* exon mutation numbers in pCR prediction was 0.711 (95% CI: 0.626–0.797) in the discovery cohort and 0.647 (95% CI: 0.533–0.762) in the validation cohort, reaching a level comparable to the ctDNA status of the whole panel (Fig. 4H). The mutational hotspots of *TP53* were shown with lollipop plots (Supplementary Fig. S4J, Supplemental Digital Content, http://links.lww.com/JS9/E32).

### Predictive models based on post-NICT TP53 mutation status and SUVmean

**Figure 5.**
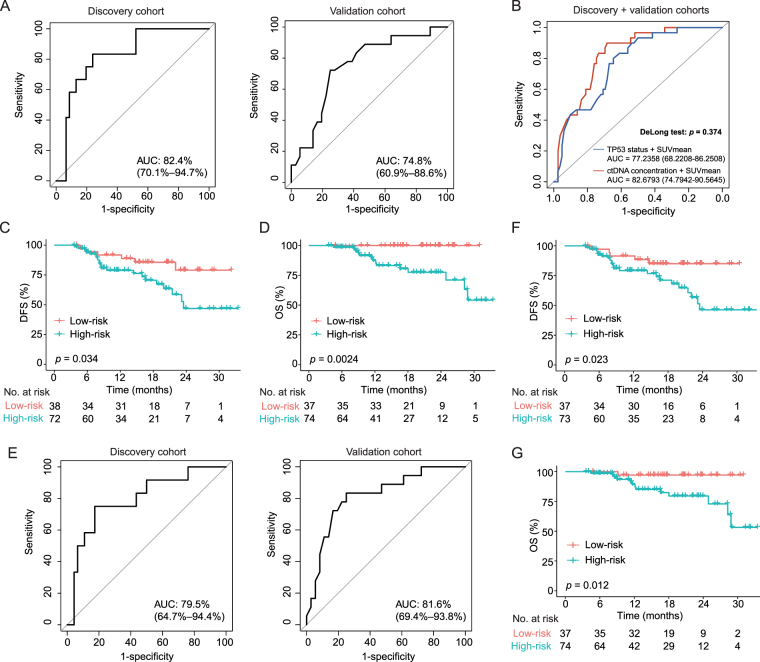
Predictive models based on *TP53* mutation status and SUVmean. (A) ROC curves reflecting the pCR predictive performance of the prediction model based on *TP53* mutation status and SUVmean in the discovery and validation cohorts. (B) The comparison of the predictive performance between the model constructed based on *TP53* mutation status and the model constructed based on ctDNA status. (C and D) Kaplan–Meier curves demonstrating the (C) DFS and (D) OS outcomes of low-risk (red) and high-risk (green) patients in the combined cohort (risk score calculated by prediction model based on *TP53* mutation status and SUVmean). (E) ROC curves reflecting the pCR predictive performance of the prediction model based on *TP53* exon mutation numbers and SUVmean in the discovery and validation cohorts. (F and G) Kaplan–Meier curves demonstrating the (F) DFS and (G) OS outcomes of low-risk (red) and high-risk (green) patients in the combined cohort (risk score calculated by prediction model based on *TP53* exon mutation numbers and SUVmean). *P* values in panel (B) were determined by the DeLong test; *P* values in panels (C), (D), (F), and (G) were determined by the Log-rank test. AUC, area under curve; DFS, disease-free survival; OS, overall survival.

As the integration of SUVmean and ctDNA concentration showed promising potential in predicting pCR status in patients, we asked whether we could replace ctDNA concentration with *TP53* mutation status, which might strikingly decrease the cost of sequencing and reduce the economic burden for patients. The logistic regression model constructed based on *TP53* mutation status and SUVmean effectively discriminated (AUC = 0.824, 95% CI: 0.701–0.947) the pCR or non-pCR status of ESCC patients (Fig. 5A). The performance of the model was verified in the validation cohort (AUC = 0.748, 95% CI: 0.609–0.886, Fig. 5A). DeLong’s test showed that there was no significant difference in predictive performance between the model constructed based on *TP53* status and the model constructed based on the ctDNA status (Fig. 5B and Supplementary Fig. S5A and B, Supplemental Digital Content, http://links.lww.com/JS9/E32). Additionally, patients with low-risk scores had longer DFS (Fig. 5C and Supplementary Fig. S5C and D, Supplemental Digital Content, http://links.lww.com/JS9/E32) and OS (Fig. 5D and Supplementary Fig. S5E and F, Supplemental Digital Content, http://links.lww.com/JS9/E32). Furthermore, the logistic regression model constructed based on *TP53* exon mutation numbers and SUVmean also discriminated the pCR or non-pCR status of ESCC patients in the discovery cohort (AUC = 0.795; 95% CI: 0.647–0.944) and the validation cohort (AUC = 0.816; 95% CI: 0.694–0.938; Fig. 5E). Based on the model, low-risk patients had longer DFS (Fig. 5F) and OS (Fig. 5G).

## Discussion

The accurate assessment of pCR is crucial for the selection of therapeutic strategies, including active surveillance, esophagectomy, or adjuvant therapy in ESCC patients who underwent NICT. Despite the clinical significance, there is a notable absence of effective non-invasive predictive approaches that can provide reliable insights into treatment responses following neoadjuvant immunotherapy^[^23^]^. In this *post hoc* analysis of clinical trials, we have, for the first time, elucidated the complementary predictive value of combining ctDNA with PET/CT in predicting the pCR to NICT for ESCC. Intersection of key gene modules converged to *TP53* as the core post-NICT ctDNA mutation for pCR prediction, and the model constructed based on *TP53* mutation and SUVmean had a comparable performance to the model based on PET/CT and the overall ctDNA concentration. By integrating these non-invasive approaches, we developed a robust predictive model that not only assessed the efficacy of the treatment but also prognosticated patient outcomes. The validation of our predictive model in an independent cohort reinforced its potential to impact clinical decision-making and patient management strategies.

Published data showed that neither endoscopic biopsy nor CT alone can accurately predict pCR^[^24,25^]^. A previous study reported that the negative and positive predictive values of regular endoscopic biopsy were 35% (95% CI: 16%–53%) and 83% (95% CI: 68%–98%), respectively^[^11^]^. Similarly, the negative and positive predictive values of PET/CT were only 44% (95% CI: 26%–63%) and 77% (95% CI: 68%–87%), respectively^[^11^]^. A pCR prediction model that incorporated post-treatment biopsy and PET/CT achieved an AUC of 0.724^[^26^]^, which is lower than the AUC (0.86) of our model integrating ctDNA and PET/CT. The synergistic predictive performance of our model after combining ctDNA with PET/CT imaging offers a nuanced understanding of the evaluation of pCR in ESCC. ctDNA, as a liquid biopsy, provides a direct measure of tumor-derived genetic material and has emerged as a sensitive indicator of tumor burden and minimal residual disease (MRD)^[^27^]^. Its high positive predictive value (PPV) is particularly advantageous as it can detect minimal residual disease and predict treatment outcomes with greater sensitivity than traditional imaging alone. A recent study found that ctDNA negativity and high blood tumor mutation burden (bTMB) levels correlated with better tumor response and survival in patients with advanced ESCC who underwent definitive chemoradiotherapy combined with toripalimab^[^22^]^. On the other hand, PET/CT is a functional imaging modality that excels in identifying areas of increased glucose metabolism, indicating the tumor activity^[^28^]^. However, its high false-positive rate due to inflammation or other non-malignant processes can confound the interpretation of treatment response^[^29,30^]^. By evaluating the minimal residual disease through ctDNA and metabolic changes via PET/CT, we offer a multi-modal strategy for predicting pCR in ESCC. Zhang *et al*. developed a pCR prediction signature using transcriptomic levels of immune-related genes in pretreatment ESCC patients^[^31^]^, and another study built a model combining small RNAs in the blood and clinical factors (including PET/CT and biopsy) that achieved an AUC of 0.84^[^32^]^. However, both models rely on biopsy and lack of prospective validation cohorts. Our prospective study validated the feasibility and clinical applicability of integrating non-invasive ctDNA and PET/CT. Advanced imaging techniques, such as ^18^F-anti-PD-L1, ^89^Zr-nivolumab, and ^89^Zr-atezolizumab as immunoPET molecular imaging agents, offer new options for evaluating tumor response to immunotherapy^[^33,34^]^. Although immunoPET is more specific than ^18^F-FDG PET/CT in distinguishing residual tumors, it has not yet been widely adopted in clinical practice^[^33-35^]^. Compared to PreSANO^[^11^]^, which focused on the pCR evaluation of neoadjuvant chemoradiotherapy, our study emphasizes the value of non-invasive assessments of pCR by employing non-invasive ctDNA and PET/CT after NICT. This comprehensive approach is particularly beneficial in settings where EUS is less accessible. As ctDNA sequencing technology advances, its predictive capabilities are expected to improve, offering a valuable minimally invasive tool for pCR prediction.

Regarding the methodological novelty, we for the first time proposed a research paradigm in investigating core ctDNA mutations by converging the whole ctDNA profiles into gene mutation modules. Given that the function of a specific mutation manifests its interaction with other proteins and its impact on treatment sensitivity, the whole ctDNA profiles were dissected into PPI-based modules and treatment sensitivity-based modules in our study. Previous studies on ctDNA merely investigated the overall concentration or status^[^15,36^]^, leading to neglect of the underlying functions of core mutations and the adulteration of confounding mutations with rare contributions to treatment response. Our research paradigm enabled us to catch core ctDNA mutations on NICT from both the perspectives of protein interaction and treatment response, which ultimately narrowed down to the *TP53* mutation. In addition, the module-resolution analysis could also overcome the dilemma of investigating some specific genes with low mutation or detection rates but share similar biological functions or participate in the same biological process, enabling a more comprehensive and deeper understanding of the ctDNA mutation profiles.

*TP53* was the dominantly mutated genes detected in ctDNA at a rate of 63%, which is similar to previous studies^[^22,37^]^. *TP53* mutations disrupt the inhibition of proliferation, migration, and invasion mediated by wild-type TP53 in ESCC and alter its protein nucleoplasmic localization and protein stability^[^38,39^]^. Our findings suggested that the detection of *TP53* mutations in ctDNA, particularly in the exons, may serve as a biomarker for predicting pCR and survival in ESCC patients who received NICT. This is supported by existing literature that links *TP53* mutations with tumor progression and therapeutic resistance^[^40^]^. Furthermore, the clearance of *TP53*-mutated cells has been associated with tumor elimination and improved therapeutic outcomes^[^41,42^]^, indicating that monitoring the changes in *TP53* mutations via plasma cfDNA is a potential approach to predict MRD or recurrence. We also found that there was no significant difference in predictive performance between the model constructed based on *TP53* status and the model constructed based on ctDNA status. The potential clinical application of our findings lies in the use of *TP53* detection in ctDNA and PET imaging to assess the risk of pCR and recurrence. This *TP53*-targeted ctDNA detection approach could reduce the need for extensive ctDNA panel testing, offering a more cost-effective and less financial burden option for patients. Further investigation into the mechanistic links between *TP53* mutations, especially in the exons, and treatment outcomes is essential for fully understanding the role of *TP53* in ESCC NICT treatment monitoring. Additionally, integrating *TP53*-targeted ctDNA detection and PET/CT predictions into the clinical decision-making process will require further research and validation.

The present study has some limitations. First, even though both the training and validation cohorts were derived from prospective clinical studies, this was a single-center study, which may introduce selection bias. Further studies involving multiple centers are required to verify the efficacy of integrating ctDNA and PET/CT in predicting the pathological response to NICT in ESCC patients. Second, the exploration of dynamic changes of ctDNA is limited. The current study primarily focused on predicting pCR, which limited our analysis to only two preoperative ctDNA assessments. Moving forward, we also conducted longitudinal postoperative ctDNA monitoring for the patients and planned to incorporate both pre- and postoperative ctDNA dynamics in our follow-up research to further investigate their roles in predicting recurrence and treatment resistance, thereby enhancing our understanding of tumor behavior and treatment outcomes. Moreover, the findings of the study are currently limited to ESCC, with the applicability to esophageal adenocarcinoma (EAC) yet to be determined. Further research is required to explore the potential of our predictive model across different esophageal cancer subtypes.

In summary, the results of this study suggest that integrated *TP53*-centric ctDNA detection and PET/CT is a useful tool for the prediction of pCR and survival outcomes of NICT in patients with ESCC. Further studies with multi-center and larger sample size would be beneficial to validate this conclusion.

## Data Availability

The data reported in this paper are available from the corresponding author upon reasonable request.

## Research registration unique identifying number (UIN)

The unique identifying numbers (UIN) are NCT04822103 (registered at ClinicalTrials.gov, https://clinicaltrials.gov) and ChiCTR2000028900 and ChiCTR2100051763 (registered at Chinese Clinical Trial Registry, http://www.chictr.org.cn/).
